# Metallurgical investigation of early failure of heater pipes in a gas complex

**DOI:** 10.1038/s41598-023-32381-2

**Published:** 2023-03-31

**Authors:** Waleed Khalifa, Shimaa El-Hadad

**Affiliations:** 1 Department of Mining, Petroleum and Metallurgical Engineering, Faculty of Engineering, Cairo University, Giza, 12613 Egypt; 2 Central Metallurgical Research and Development Institute, P.O. 87, Helwan, Egypt

**Keywords:** Materials science, Metals and alloys

## Abstract

An early corrosion failure of the piping system of a gas heater was reported by a gas complex company. Local corrosion rates of 0.90 and 0.66 mm/year were observed in the heater piping system. An investigation including visual examination, macrostructure, microstructure (SEM, EDS and XRD), thickness gauging, chemical analysis, and mechanical testing, was performed. The results showed that corrosion damage occurred on the external surface of the pipes. Corrosion occurred at the cold sides of the pipes and elbows. The corrosion pattern is broad and shallow pitting. Some elbows showed an early stage of carbide spheroidization and pearlite decomposition. The EDS microanalysis revealed that the level of sulfur, chlorine, and nitrogen was substantially high in the rust samples. The XRD of the corrosion products showed that the main oxyhydroxide was Akaganeite. The analysis of results showed that the flue gas dew point corrosion was the mechanism of damage, and the root cause was the operation of the heater at low temperatures and the frequent outings of service, combined with evident material drawbacks including low levels of Si, Cu, Ni, Cr, and Mo. These elements should be at their maximum allowable limits of the SA 105 and SA 106 to improve the corrosion resistance of the steel piping components.

## Introduction

Failure of natural gas heater pipes causes thinning of the pipes and possibly local loss of the whole pipe material^1^. This can lead to frequent shutdown and even explosions in the worst cases. One of the most common causes of failure is corrosion^2^. Majid et al.^3,4^ in their analysis of gas heater pipes observed that failure first occurs by erosion of the pipe under the action of a water jet that comes from a nearby leaking water pipe and got mixed with erosive particles from the surrounding soil. As a result, the pipe coat is locally removed and the corrosion process coincides.

Wang et al.^5^, in their recent investigation of an elbow (45°) in a pipeline system that gathers natural gas, observed a change in the radial of the fluid state. They owed this to the low velocity of the flow, the high pressure, and the turbulence in the kinetic energy. All these factors allowed the water vapor containing CO_2_ to condense; these condensed droplets then flowed back by the gravity action and agglomerated at the position of connection between the horizontal pipeline and the elbow, thus causing electrochemical corrosion of the elbow.

**Figure 1:**
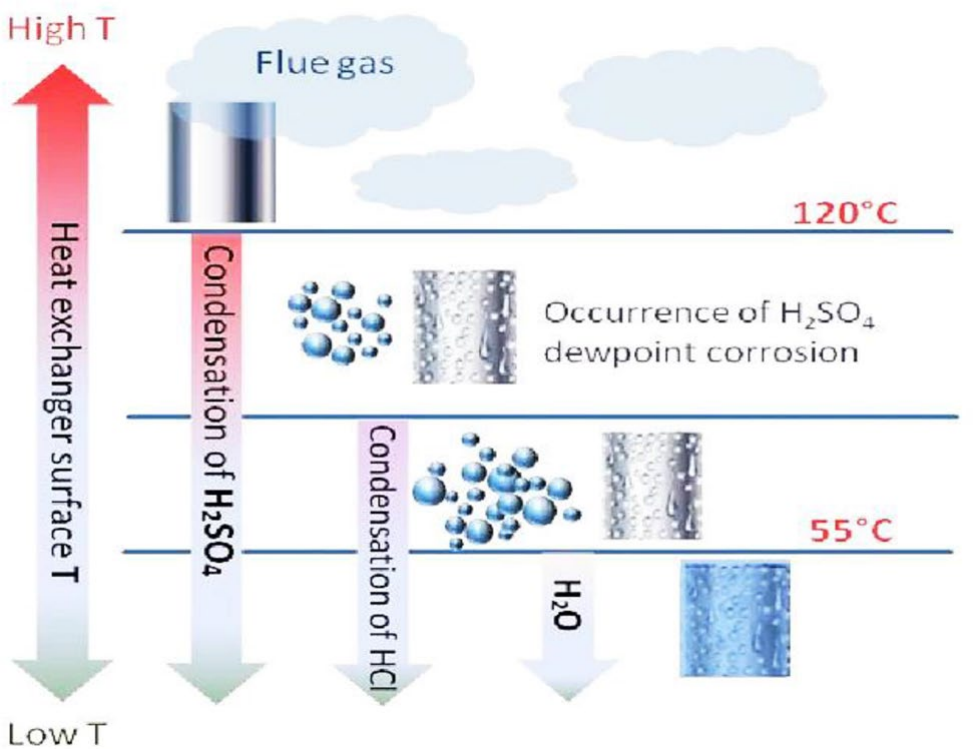
A schematic illustration of “dew point corrosion” mechanism in heater exchangers^8^.

Another mechanism of gas heater pipe failure is the so-called dew point corrosion, which is very serious, especially in carbon steel, alloy steel, and stainless steel. Dew point corrosion occurs due to condensing acid gases such as hydrogen chloride, sulfur dioxide, sulfur trioxide, and other combustion products. This corrosion occurs solely when the metallic component’s surface temperature falls below the dew point of the flowing gas^6,7^. In this case, an aggressive corrosion attack occurs and leads to perforation or even rupture of the components. A schematic presentation of the “dew point corrosion mechanism is shown in Fig. 1^8^.

In the current work, an early corrosion failure occurred in a heater piping system of a gas complex. The provided information mentioned that the service life of the heater was 10 years. Leakage due to perforation was observed during the shutdown in the summer. The heater receives dual-phase gas at about 56 °C and produces a single-phase gas at about 60 °C. The major part of the heat provides latent heat for liquid droplets to transform into gas phase. The heater accordingly, raises the gas temperature from 4 to 10 °C. Dry natural gas is used as fuel for the heater. The heater frequently goes out of service because of the operation conditions. Detailed microstructure studies of the failed parts along with corrosion tests were done. The root cause of corrosion damage in the system was revealed and recommendations to avoid future damages were suggested.

## Methodology of investigation

**Figure 2:**
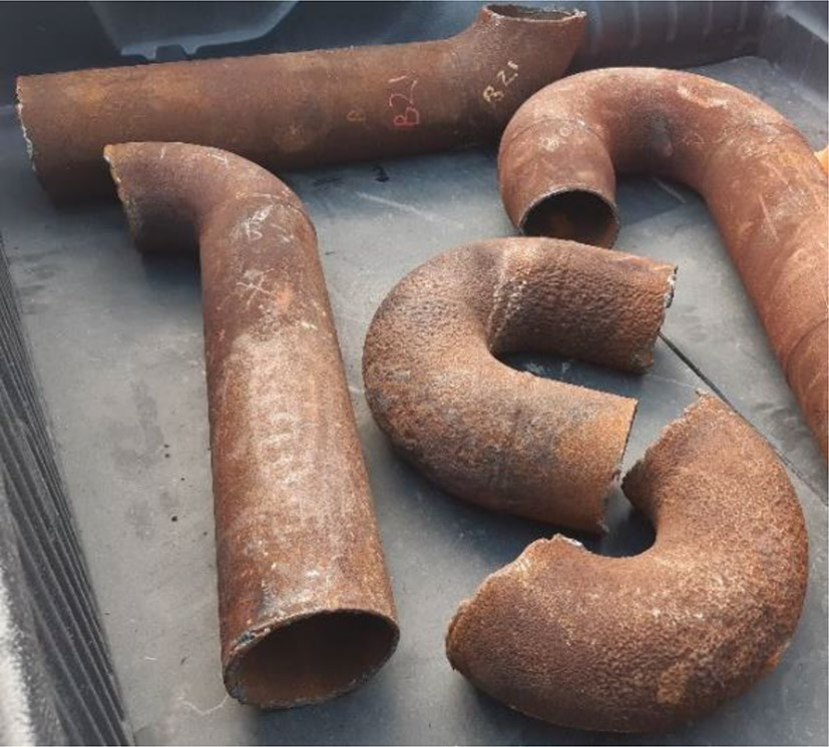
Investigation samples.

To reveal the root cause of the early failure of the pipe, some samples were selected as shown in Fig. 2. A visual examination was followed by a detailed microstructure study and corrosion tests of the failed specimens were performed.

### Visual examination

**Figure 3:**
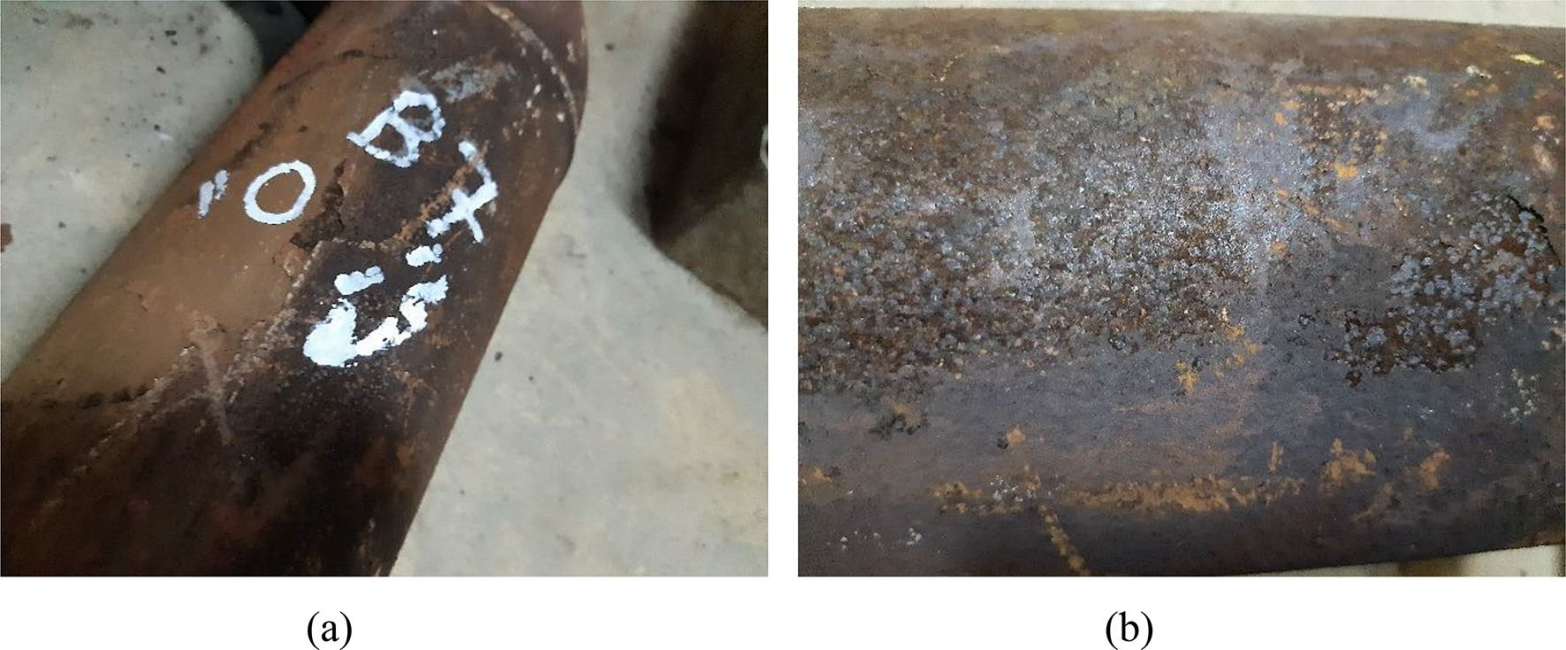
Images of the damaged pipe B7: (**a**) no corrosion at the hot side of the pipe facing the burner, and (**b**) slight corrosion at the cold side of the pipe facing the shell. The pipe was at the lower part of the radiant section of the heater. Slight corrosion damage occurred at the cold side of the pipe facing the shell. The damage is shallow pitting.

**Figure 4:**
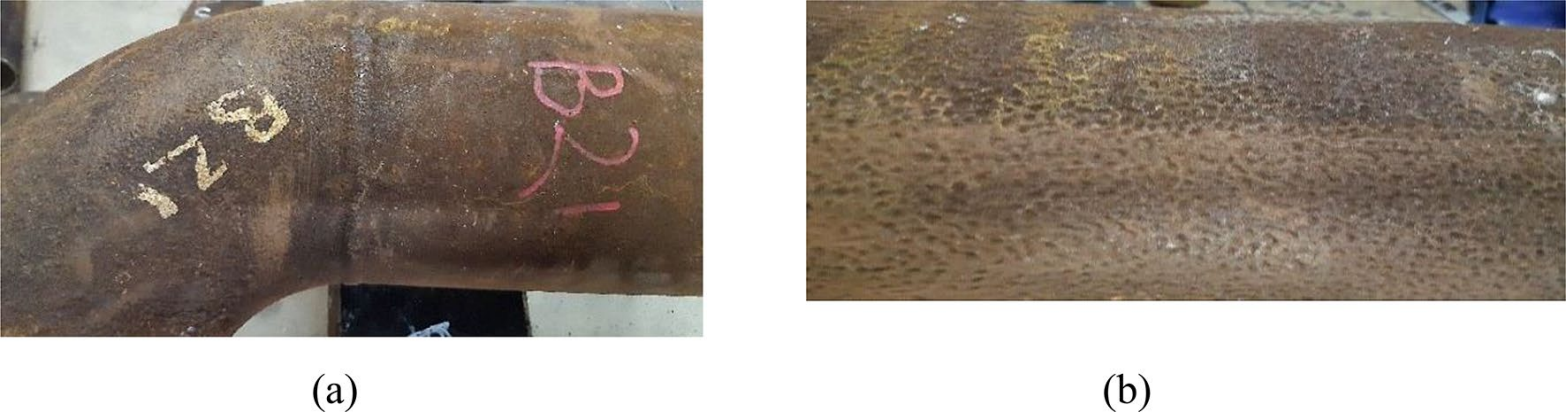
Images of the damaged pipe B21: (**a**) no corrosion at the hot side of the pipe facing the burner, and (**b**) slight corrosion at the cold side of the pipe facing the shell.

**Figure 5:**
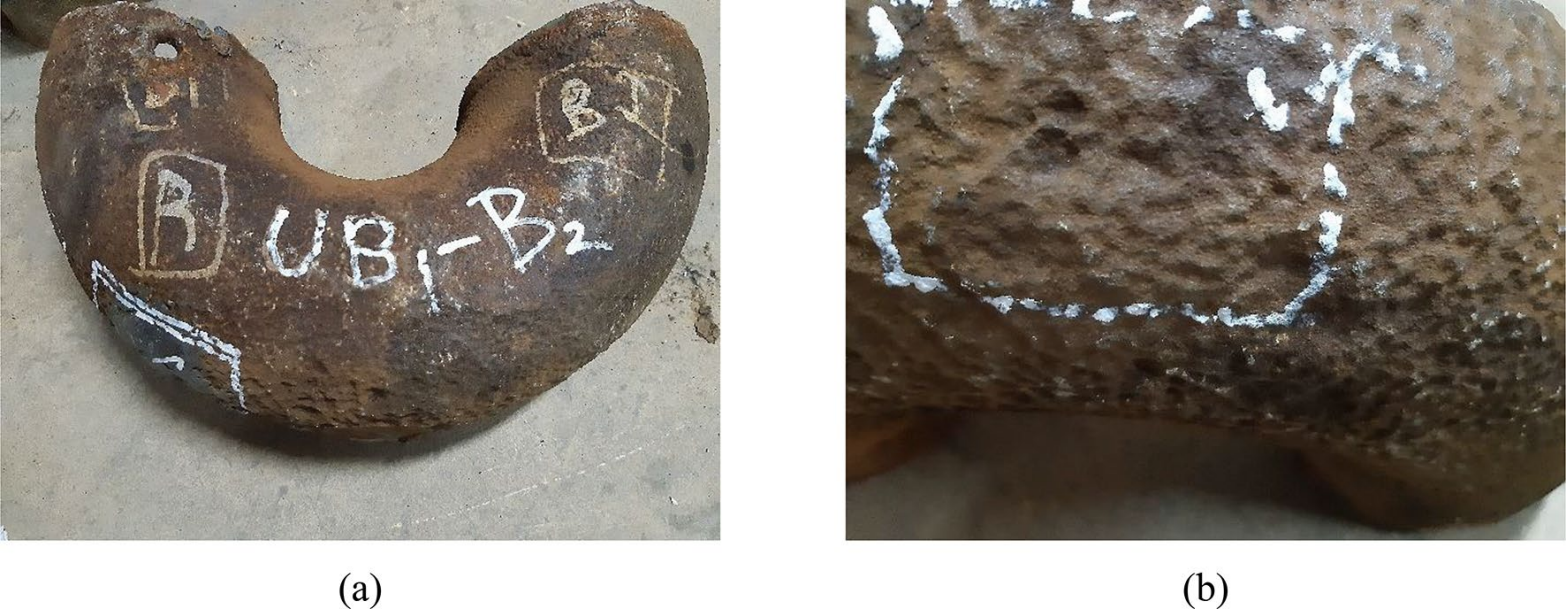
Images of the damaged elbow UB1–B2. (**a**) no corrosion on the hot side facing the burner, (**b**) severe corrosion at the U bend and the cold side facing the shell. The elbow was at the lower part of the radiant section of the heater, and the U bend was down in service. Corrosion damage occurred at the bottom of the elbow and on the cold side facing the heater shell. The damage is coarse and shallow pitting.

**Figure 6:**
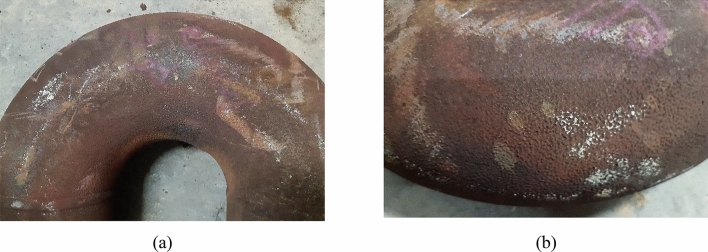
Images of the damaged elbow UB10–B11: (**a**) side of the elbow, and (**b**) bottom of the elbow. The elbow was at the lower part of the radiant section of the heater, and the U bend was down in service. Slight corrosion damage occurred at the bottom of the elbow. The damage is shallow pitting.

**Figure 7:**
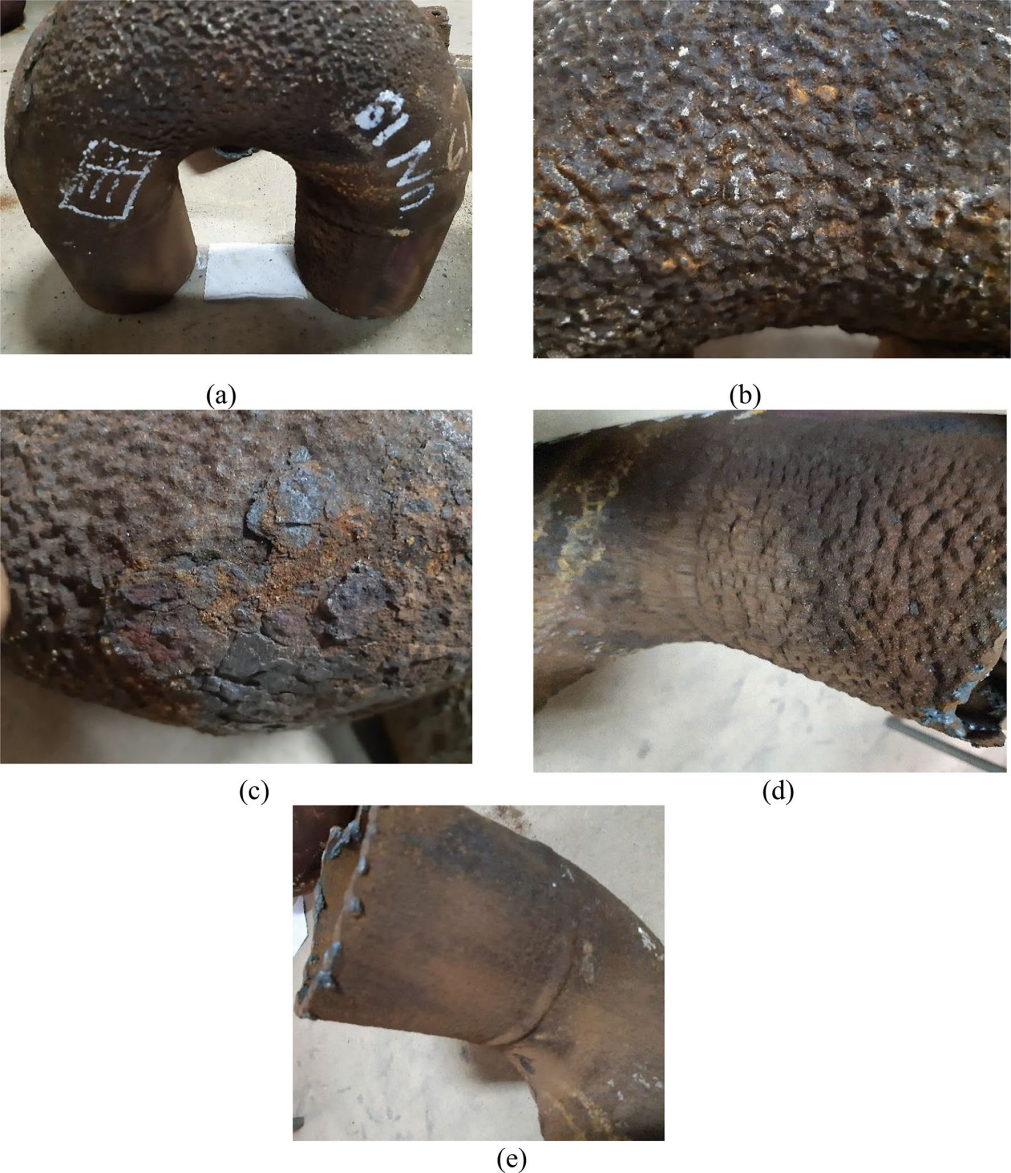
Images of the damaged elbow UN19. (a) general views, (b) coarse and shallow pits, (c) flaky and coarse gained rust layers, might indicate the increased chloride contamination in the rust, (d) lower leg covered with deposits, and (e) upper leg not covered with deposits. The elbow was at the convection section of the heater on the north side, and the U bend was facing the north direction, which is the dominant direction of marine and coastal winds. Corrosion damage occurred at the U bend, and the elbow leg was covered with deposits and sediments in service. The damage is coarse and shallow pitting.

The visual examination aims at describing the state of damage and the morphology of the failed parts. Figures 3, 4, 5, 6, and 7 show surface photographs of the investigated piping components. By reviewing these figures, it is clear that the corrosion damage is most severe in the elbows UN19 and UB1–B2 (Figs. 5 and 7). B7, B21, and UB10–B11 show minimal corrosion damage (Figs. 3, 4, and 6). The general observation of this examination is that the corrosion damage occurred at the external surface of the pipes rather than the internal surface which was sound and intact. This indicates that the damage happened because of the reaction of the heater’s internal atmosphere (i.e., flue gases) with the steel piping of the coil. The major and most important observation is that corrosion occurred on the cold sides of the pipes and elbows. The corrosion pattern is broad and shallow pitting.

The location of damages was mainly at the U bends of the elbows at the bottom of the heater. For the elbow UN19 (Fig. 7), severe corrosion occurred at the U bend facing the dominant marine and coastal winds. These observations give an insight into damage related to condensation of water or liquids on the cold piping areas, such as piping sides facing the shell and cold areas at the lower parts of the piping system where water and liquid droplets would gather because of gravity.

It is worth mentioning here that UB1–B2 was at the beginning of the heater coil, which receives first the cold gas for processing. So, it is the coldest pipe and elbow in the heater. Thus, it is not strange to be heavily damaged and the first pipe to perforate in the heater.

The summary of the visual examination is that:

- The corrosion damage occurred at the external surface of the pipes, indicating a reaction between the heater’s internal atmosphere (i.e., flue gases) and the steel piping system.
- The internal surfaces of pipes and elbows were sound and intact.
- Corrosion occurred at the cold sides of the pipes and elbows facing the heater shell.
- The corrosion pattern was broad and shallow pitting.
- The damage was due to condensation of water or liquids on the piping system.

### Macrostructure

**Figure 8:**
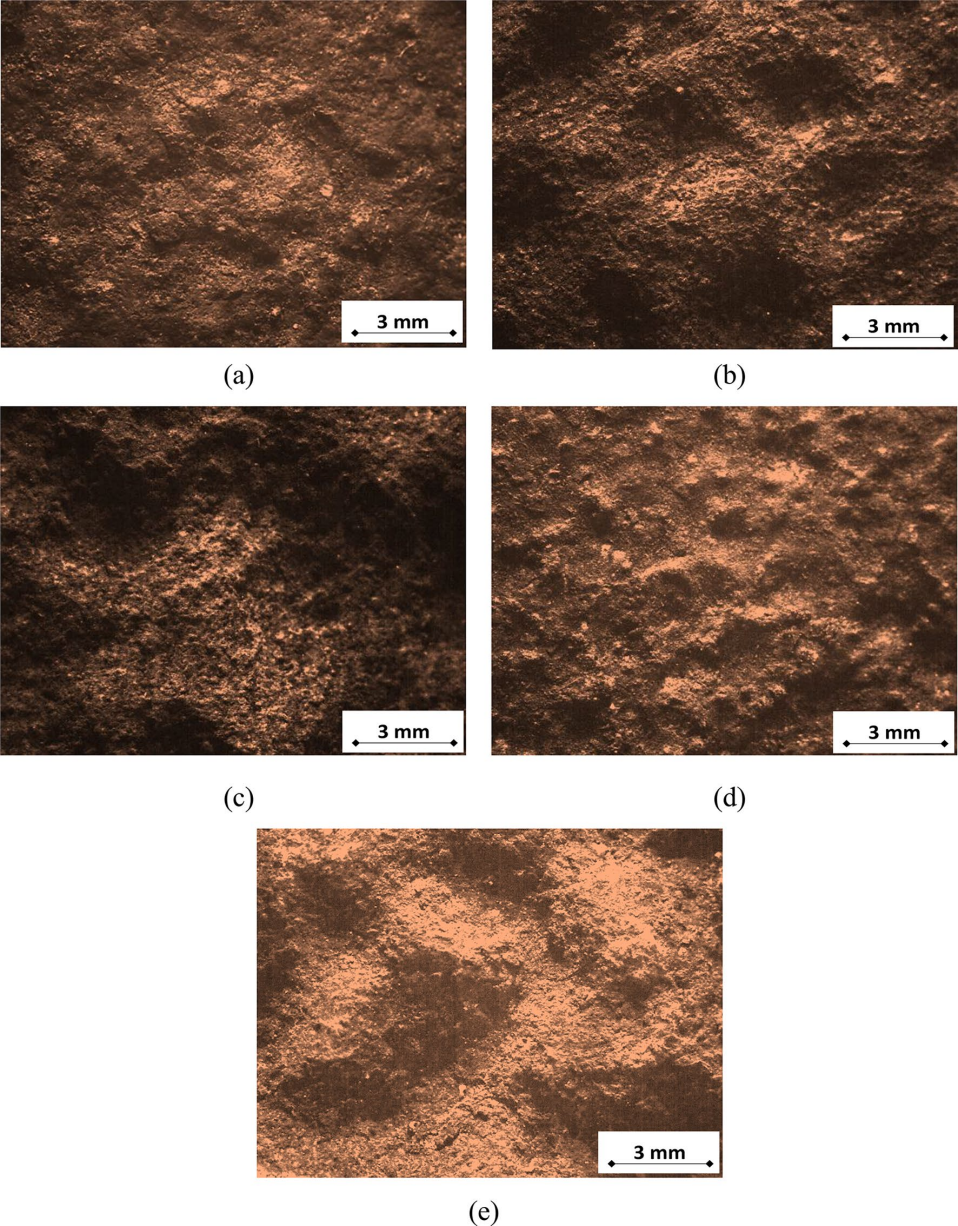
Macrographs of the external corrosion of (**a**) pipe B7, (**b**) pipe B21, (**c**) elbow UB1–B2, (**d**) elbow UB10–B11, and (**e**) Elbow UN19.

Compared to visual examination, the stereoscopic study of the macrostructures is always done to give more details of the failure since it is done using magnifications that range between 10 and 70 ×^9^. A Stereoscope model “Leica” was used to study the macrostructure of the failed parts. Figure 8 shows the micrographs of the external corrosion of the investigated pipes and elbows. The localized thickness loss in a pattern of broad and shallow pitting is typical of the flue gas dew point corrosion. This is clearly evidenced in Fig. 8b,c,e. The pit sizes of UN19 and UB1-B2 average at ~ 10 mm in diameter. The occurrence of corrosion pits was mainly found at the cold side of pipes and elbows (the sides facing the heater shell) and at the bottom of elbows, and in the areas covered by deposits and sediments. The corrosion of areas covered by deposits was observed during the heater inspection. These are characteristics of the flue gas dew point corrosion^5^, hence suggesting the damage mechanism for the heater piping system.

### Microstructure

**Figure 9:**
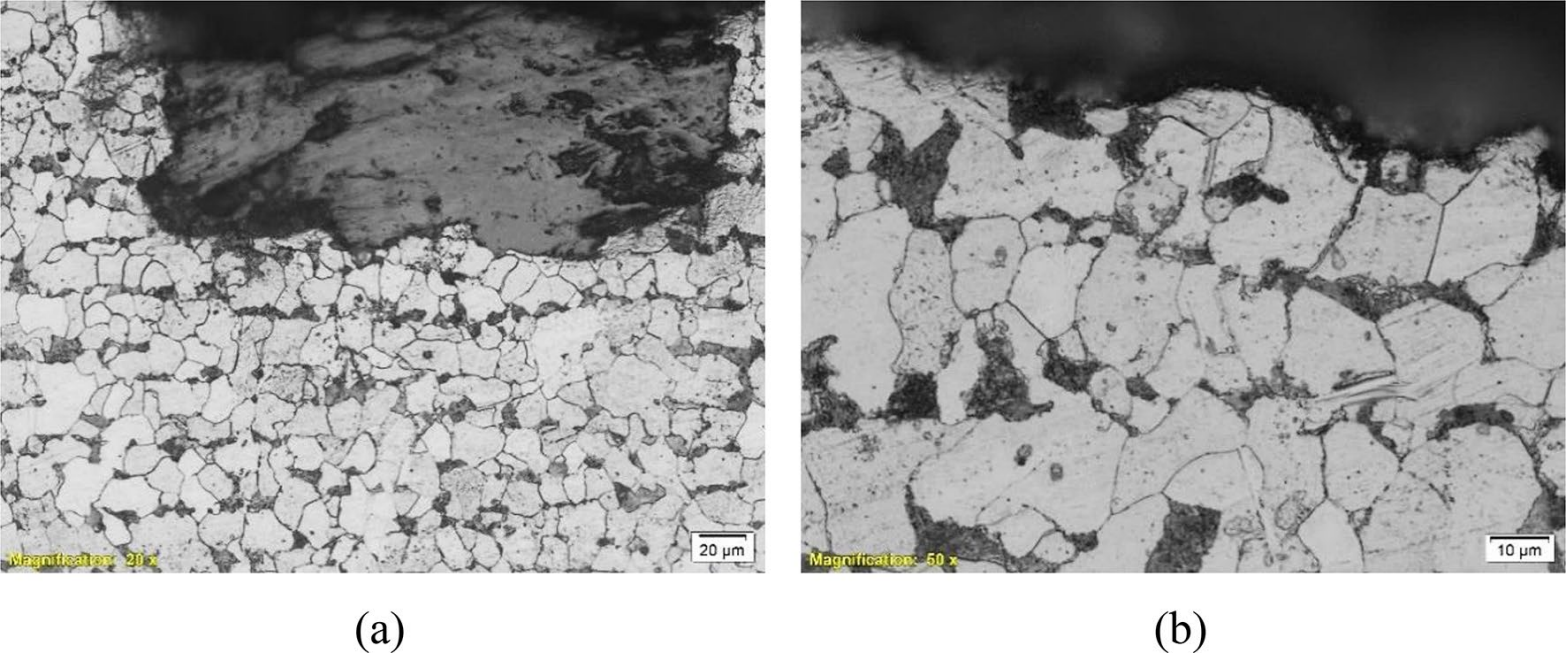
Microstructure of the failed pipe B7: (**a**) cross-section at the external corroded surface and (**b**) higher magnification showing typical carbon steel microstructure.

**Figure 10:**
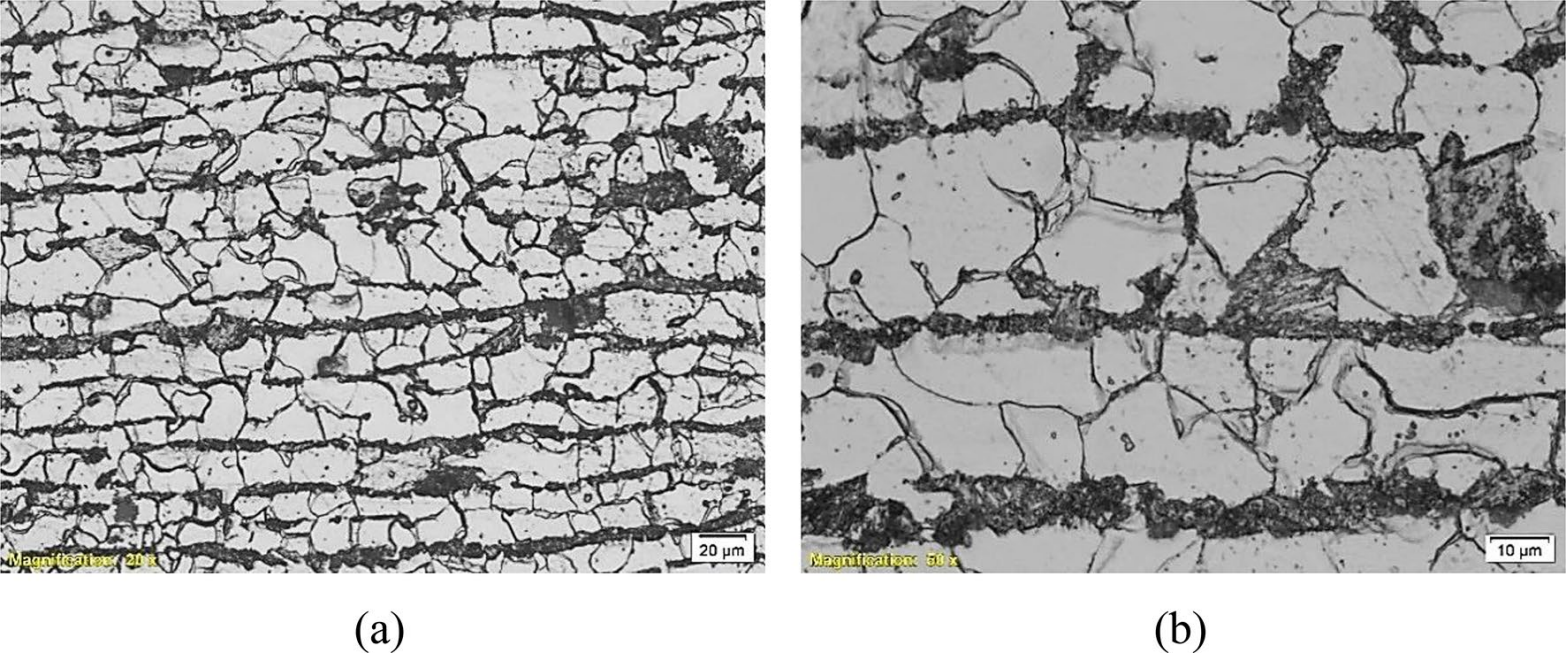
Microstructure of the failed elbow B21: (**a**) cross-section at external corroded surface and (**b**) higher magnification showing typical microstructure of carbon steel.

**Figure 11:**
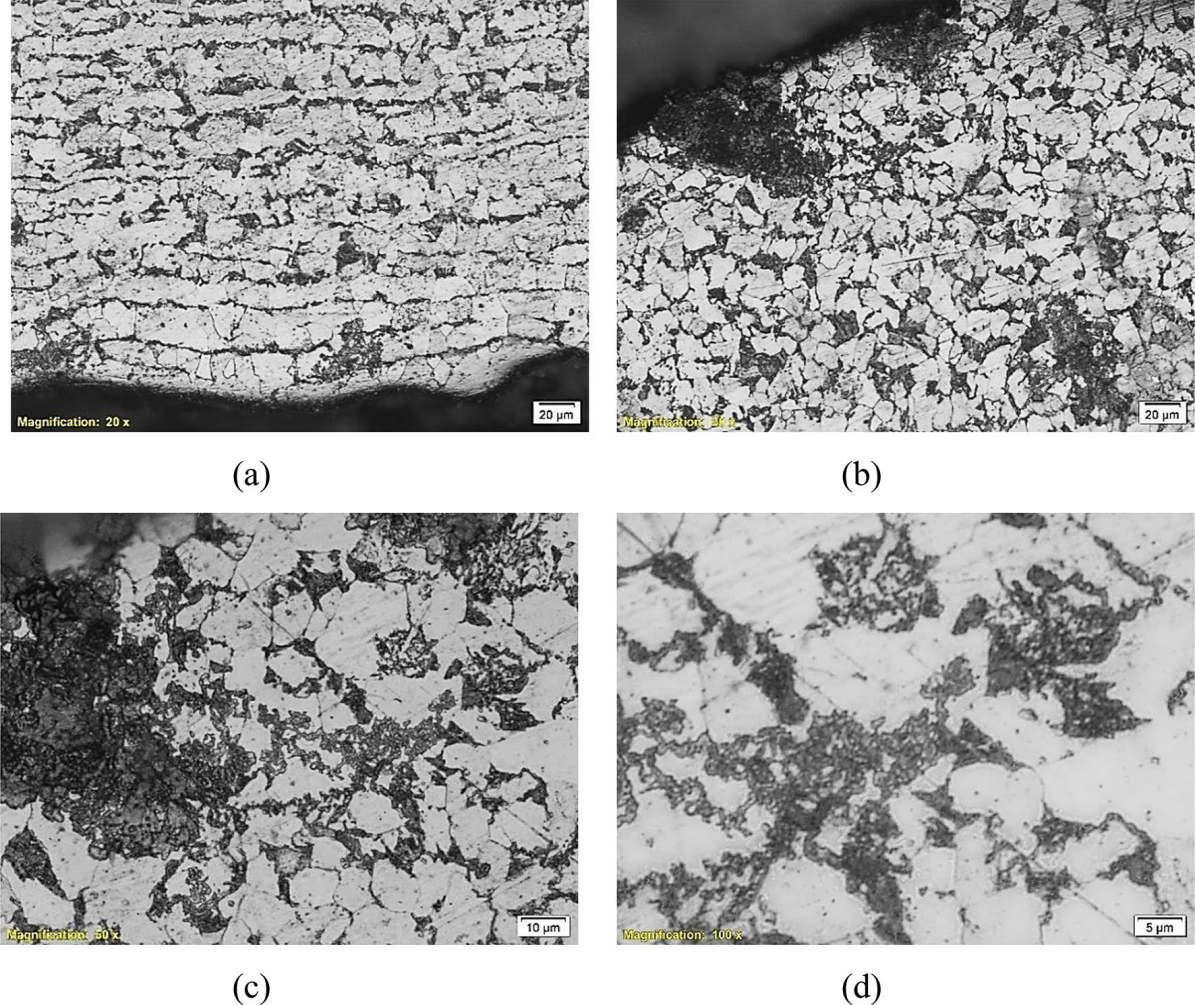
Microstructure of the failed elbow UB1-B2: (**a**) cross-section at internal surface showing typical carbon steel microstructure, (**b**, **c**) cross-section at external corroded surface, showing corrosion pits and subsurface reaction products, and (**d**) a higher magnification micrograph showing an early stage of pearlite decomposition and carbide spheroidization. This is also seen in (**a**) and the internal surface.

**Figure 12:**
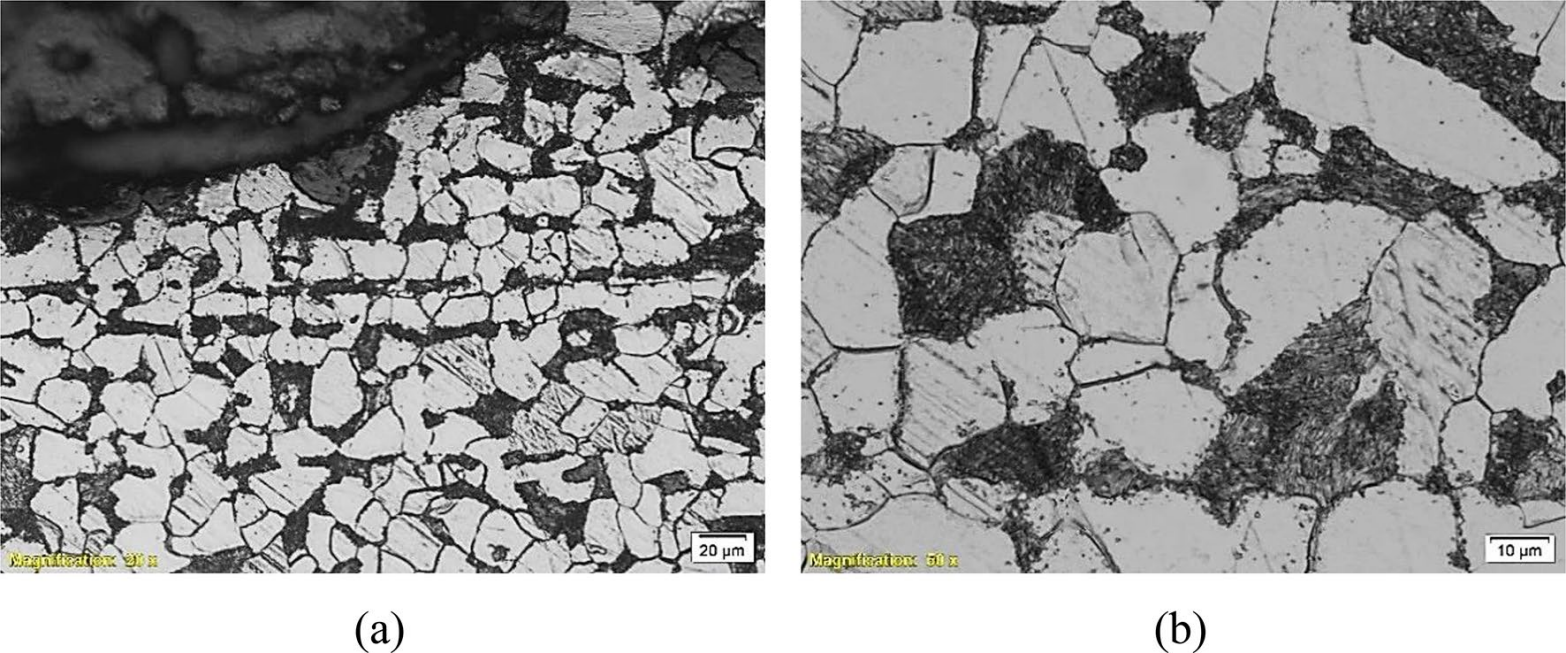
Microstructure of the failed elbow UB10-B11: (**a**) cross-section at external corroded surface, and (**b**) higher magnification view showing typical microstructure of carbon steel.

**Figure 13:**
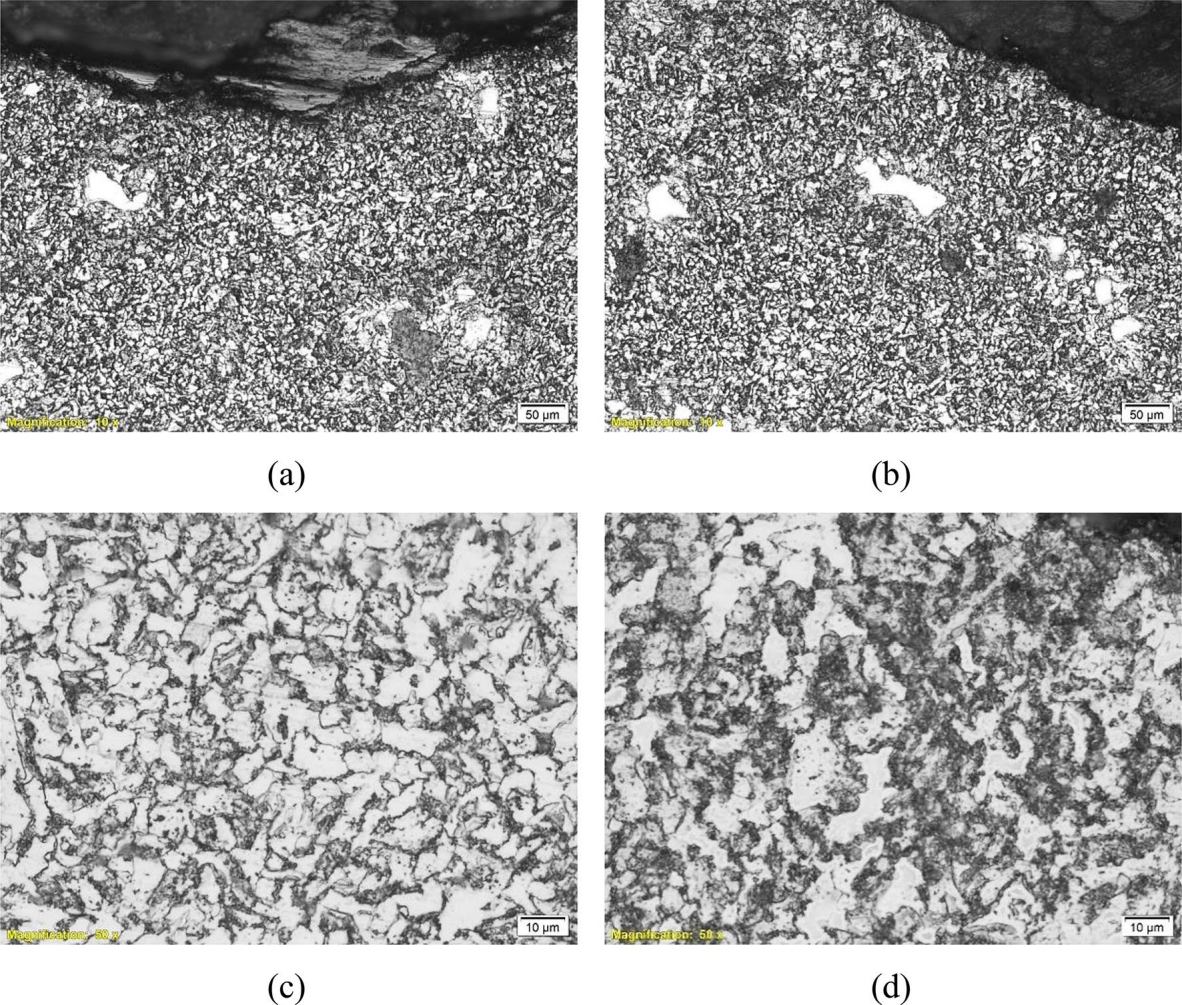
Microstructure of the failed elbow UN19: (**a**, **b**) cross-section at external corroded surface showing large areas of ferrite (white islands) and large level of pearlite (dark areas), and (**c**, **d**) larger magnification showing spheroidized carbides or partially transformed/decomposed austenite microstructure.

To deeply investigate the microstructures of the failed parts and determine whether they participated in the failure or not, an optical microscope “Olympus BX51” was used for metallographic investigation. Figures 9, 10, 11, 12 and 13 show the microstructure of the investigated pipes and elbows. The microstructures are typical of mild carbon steel*.* Banded structures are clear in B21, UB1-B2 and UB10-B11 (Figs. 10, 11, 12). The only two odd microstructures of UB1-B2 and the UN19 (Figs. 11, 13). The UB1-B2 (Fig. 11) shows corrosion pits and subsurface reaction products that indicate a chemical attack (probably chloride or sulfide attack). Both of elbows UB1-B2 and the UN19 show an early stage of carbide spheroidization (and partially decomposed pearlite). UN19 exhibits a microstructure likely produced by holding at the inter-critical temperature range between A_C1_ and A_C3_, rather than holding above A_C3_ during normalizing. It is worth mentioning that both of UB1–B2 and the UN19 show the most severe corrosion damage and deep course pitting among the five samples investigated in the current work.

These findings indicate improper manufacturing practices regarding heat treatment and or the temperature of forging. These elbows are manufactured by forging according to the SA 105 specification^10^. This is usually a hot forging process. Though the actual manufacturing conditions were unavailable, the possible scenarios of pearlite decomposition beyond the available data were called. These reasons are generally holding the steel at high temperature and for a long time. These are two factors: holding time and holding temperature. Holding the metal at a higher temperature produces pearlite decomposition at shorter times and vice versa. The wrong treatment here was believed to be mainly by holding at a higher temperature rather than holding for a long time since industry timing is likely more critical because it is directly related to daily or weekly production rates.

This faulty manufacturing does not include all the piping components of the heater as seen in this section, but only the elbows UB1–B2 and the UN19. The faulty manufacturing would participate in part in the corrosion failure, but might not be the major cause of failure.

### SEM and EDS microanalysis

**Figure 14:**
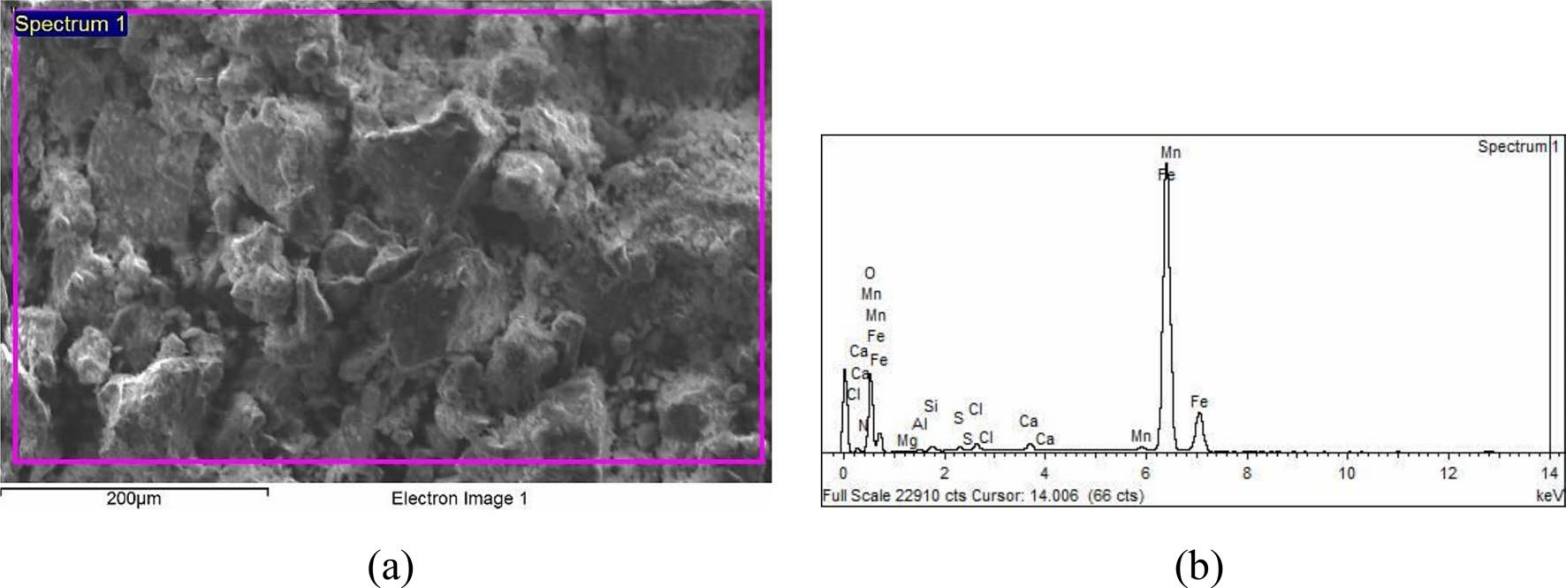
SEM backscattered image and EDS microanalysis of the corrosion products of UB1-B2: (**a**) SEM micrograph of the corrosion product, (**b**) the EDS analysis pattern 1.

**Figure 15:**
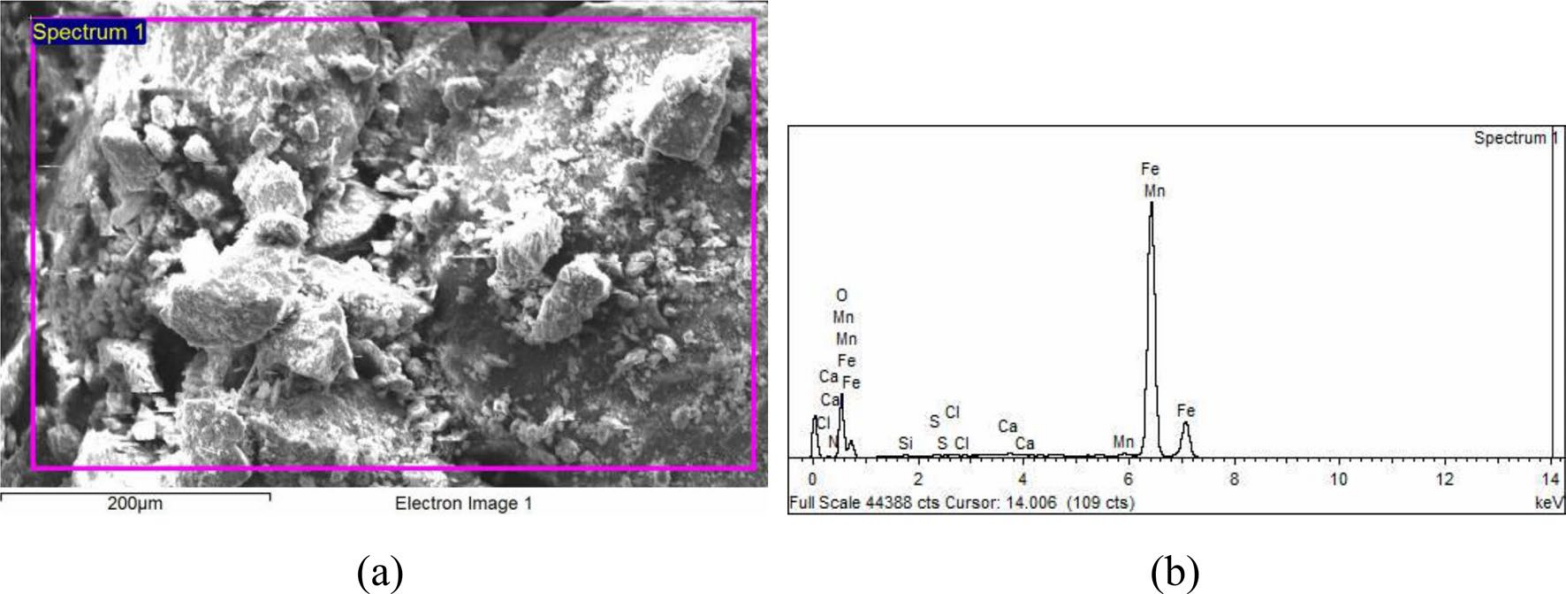
SEM backscattered image and EDS microanalysis of the corrosion products of UN19: (**a**) SEM micrograph of the corrosion product, (**b**) the EDS pattern of analysis 1.

Scanning Electron Microscopy (SEM; JEOL), combined with an Energy Dispersive X-ray Spectroscopic (EDS) was used for further microstructure investigation. According to Freeman^9^, EDS is considered one of the most common techniques for analyzing corrosion deposits. Figures 14 and 15 show the backscattered micrographs and microanalysis of the corrosion products of elbows UB1-B2 and UN19. The summary of the EDS microanalysis is shown in Table 1.

**Table 1:** EDS elemental analysis at different locations across the corroded thermowell section.

Element	Wt%				
	UB1–B2			UN19	
	Analysis 1	Analysis 2	Analysis 3	Analysis 1	Analysis 2
N	00.98	–	–	02.19	02.58
O	34.13	34.03	33.64	32.31	35.79
S	00.37	00.28	00.32	00.17	00.13
Cl	00.72	00.64	00.48	00.18	00.24
Fe	61.00	61.64	61.48	63.99	59.50

It is clear from the elemental microanalysis of the corrosion products that high levels of sulfur and chlorine occur in both elbows, UB1–B2 and UN19. These elements confirm the role of flue gas dew point corrosion in these failures. This mechanism of damage occurred when the flue gases contain contaminants of sulfide and chloride ions. At specific dew points, solutions of HCl and H_2_SO_4_ condense on the surface of the steel components and cause corrosion damage. The nitrogen ions, which also are analyzed in these corrosion products, do also accelerate the rate of corrosion damage. The severity of the damage depends on the level of contaminants and the temperature of the steel surface. The levels of sulfur, chlorine and nitrogen are substantially high in the rust samples. The permissible levels of S and Cl in steels are almost zeros. For example, the S level must be at a max of 0.035 wt% in existing steel pipes as can be seen in Table 3 of the manuscript. The Actual level of S in the corroded pipes as shown in Table 3, is 0.004 wt%. Thus, the level of S which was analyzed in the corrosion products (between 0.13 to 0.37 wt%) is considered high and thus played a role in the observed damage. The Cl is not an alloying addition to steel, nor it is a tramp element in the steel raw materials, and can only be deposited on the coil pipe surface from the atmosphere. The observed level of chlorine (chlorinity) was between 0.18 to 0.72 wt%. The salinity level is thus, = 1.80655 × Chlorinity = between 0.33 to 1.3 wt %. This is a high level and would contribute to the observed corrosion.

### XRD analysis

**Figure 16:**
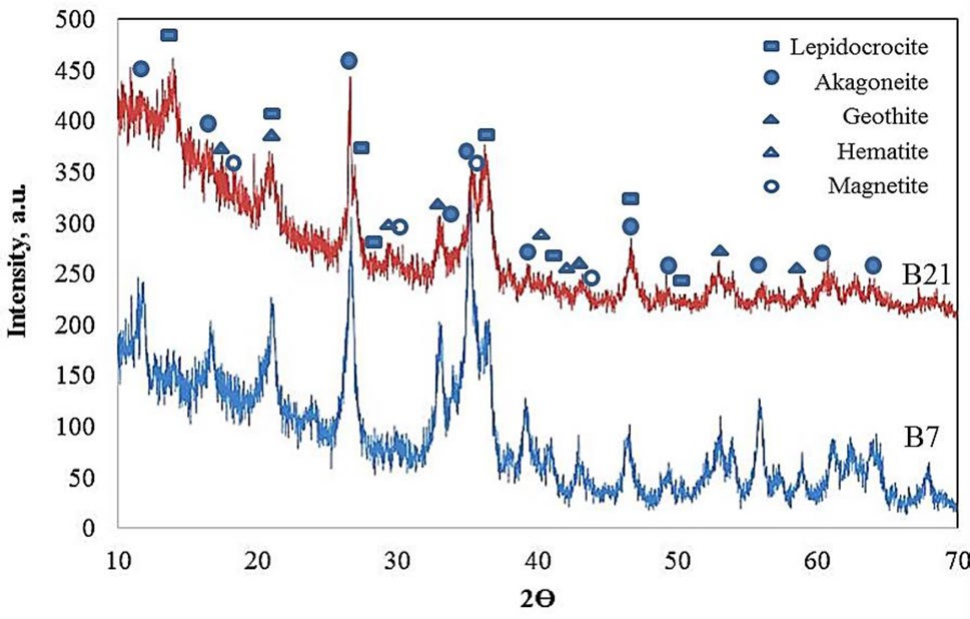
XRD diffraction patterns of selected failed samples.

To identify the different compounds that formed upon corrosion, corrosion products were collected from the surface of the pipes and elbows and analyzed using an X-ray Fuorescence (XRF) analyzer “Axios advanced-PANALYTCAL, The Netherlands”. Examples of the X-ray diffraction patterns are shown in Fig. 16. The rough distribution of the detected compounds is also presented in Table 2. The main ferric oxyhydroxides (i.e., Lepidocrocite, Akaganeite, and Goethite), and iron oxides (i.e., Hematite, Magnetite and Wustite\Plustite) are shown with their relative levels in the corrosion products. For the pipes and elbows located inside the heater which are exposed to the flue gases (B7, B21, UB1–B2, and UB10–B11), the main oxyhydroxide which occurred is the Akaganeite. The Lepidocrocite and the goethite occurred at lower levels while the Akaganeite occurs only in the chloride-laden coastal and marine environments^11^, which is the current case. In the presence of chloride ions other ferrous, ferric and magnetite oxides can form. The driving for these reactions is the presence of chloride/saline deposits and the pollutants of SO_x_ and NO_x_ in air^12^. The dominance of Akaganeite inside the heater indicates to the presence of chlorides in the flue gases. Contaminants, such as SO_2_ and NO_xs_ might also be contained in the flue gases. The origin of these corrosive matters might be either resulting from the combustion of the fuel gas, or coming with air used for combustion.

**Table 2:** Analysis of XRD patterns for the investigated samples.

Sample	Compounds					
	Lepidocrocite FeO(OH)	Akaganeite FeO(OH)	Goethite, FeO(OH)	Hematite Fe_2_O_3_	Magnetite Fe_3_O_4_	Wu-/plu-stite FeO
B7	26%	52%	22%			
B21	24%	36%	10%	9%	21%	
UB1–B2	13%	27%	13%	13%	24%	10%
UB10–B11	26%	33%	14%	26%		
UN19	13%	4%	12%	21%	50%	

On the other hand, the corrosion products of the elbow UN19 of the external convection section contained much lower levels of Akaganeite. Instead higher levels of magnetite and hematite occurred in the rust. This part of the heater is not exposed to the flue gases. It is at the north side of the convection section, and might be exposed to coastal winds, which are rich in saline and chloride deposits. The interference of chloride ions with the rust-forming process leads to the formation of coarse and flaky rust layers rich in magnetite and hematite, and the chloride ions segregate at the active anode front. The UN19 sample investigated here contained mainly coarse and flaky rust layers (Fig. 7b,c). This would explain the high levels of Magnetite and Hematite in the rust of UN19.

### Chemical analysis

The chemical compositions of the investigated pipes and elbows are shown in Tables 3 and 4 as analyzed by an optical emission spectrometer (Foundry Master Pro, Germany). As can be seen from Table 2, the pipe compositions conform to that of SA-106 grade B^13^. However, the UB10-B11 elbow does not conform to the specified composition of the SA-105 because of the low level of Mn. The chemical composition of elbows UB1-B2 and UN19 conform to the SA-105 specification^10^. The level of Si is noticeably low in all the piping components, i.e., between 0.17 and 0.22%. Si is one of the elements that improve the corrosion resistance of carbon steels. Therefore, these piping components might lack part of the corrosion resistance in view of their low Si levels. Not only the Si, other elements such as Cu, Ni, Cr and Mo exist at very low levels. These elements should be at their maximum allowable limits of the SA 105 and SA 106 to improve the corrosion resistance of the steel piping components. To summarize this section, it is can be mentioned that the chemical composition of the steel pipes and elbows is not the best for the corrosive conditions of the heater.

**Table 3:** Chemical composition of the investigated pipes.

Sample	Composition, %											
	C	Si	Mn	P	S	Cr	Cu	Mo	Ni	V	Al	Comment
B7	0.13	0.22	0.66	0.016	0.004	0.11	0.27	0.03	0.12	0.00	0.03	Acceptable
B21	0.15	0.17	0.90	0.014	0.003	0.12	0.31	0.01	0.06	0.00	0.03	Acceptable
SA-106 Grade B	0.30 max	0.10 min	0.29– 1.06	0.035 max	0.035 max	0.40 max	0.40 max	0.15 max	0.40 max	0.08 max	–	

The sum of Cu, Ni, Cr, Mo and V shall not exceed 1.00%.

**Table 4:** Chemical composition of the investigated elbows.

Sample	Composition, %											
	C	Si	Mn	P	S	Cr	Cu	Mo	Ni	V	Al	Comment
UB1–B2	0.16	0.20	0.80	0.015	0.005	0.12	0.26	0.04	0.10	0.00	0.00	Accepted
UB10–B11	0.26	0.21	0.47	0.012	0.005	0.03	0.01	0.00	0.04	0.00	0.00	Rejected
UN19	0.16	0.20	0.81	0.015	0.004	0.12	0.26	0.05	0.10	0.00	0.00	accepted
SA-105	0.35 max	0.10–0.35	0.60–1.05	0.035 max	0.040 max	0.30 max	0.40 max	0.12 max	0.40 max	0.08 max	–	

The sum of Cu, Ni, Cr, Mo, V shall not exceed 1.00%. The sum of Cr and Mo shall not exceed 0.32%.

### Hardness and tensile results

Hardness was measured using a Rockwell hardness test type. A “Shimadzu” tensile machine was used for tensile tests. An average of three readings was considered. Tables 5 and 6 show the results of the hardness and tensile tests respectively. Both the hardness and tensile properties are in conformity with the specified levels. The mechanical properties of the pipes and elbows are acceptable and do not constitute a cause of failure.

**Table 5:** Hardness measurement of elbows.

Tube/pipe	HB					Average
UB1–B2	172	154	162	157	180	165
UB10–B11	154	168	160	153	171	161
UN19	172	179	167	159	177	171
SA-105	137–187

**Table 6:** Tensile properties of pipes.

Pipe	Yield strength, MPa	Tensile strength, MPa	Elongation, %	Comment
B7	344	467	24	Acceptable
B21	313	469	28	Acceptable
SA-106 Grade B	240 min	415 min	19	

### Thickness gauging

Thickness gauging was measured using an ultrasonic corrosion gauge “Olympus MG2-DL” with dual element transducer type “D790-SM”, calibrated on a steel step-wedge between 2 and 10 mm. The device accuracy is 0.01 mm. Table 7 shows the ultrasonic wall thickness measurements of the investigated piping components of the heater. It is clear from the measurements that the component wall thickness exhibited substantial variation from the original wall thickness. The variation of thickness is seen at several random locations, where local metal loss is more evident.

**Table 7:** Thickness measurements at the corroded area.

Component	UT thickness gauging Mm					Original wall thickness	Min. thickness
B7	4.4	4.4	4.5	4.5	4.4	6.55 mm	4.4
B21	4.5	4.3	4.4	4.3	4.5	7.11 mm	4.3
UB1–B2	2.9	2.6	6.4	5.9	0.0	6.55 mm	2.6
UB10–B11	9.0	8.9	9.0	9.0	8.9	7.11 mm	8.9
UN19	3.0	2.4	2.9	6.5	6.1	6.55 mm	2.4

The minimum wall thickness reached 2.4 out of 6.55 mm in elbow UN19*.* Elbow UB1-B2 was perforated by corrosion from 6.55 mm thickness in 10 years of service.

### Corrosion rate

Considering the original wall thickness of the elbow that is 6.55 mm, the measured final wall thickness of the failed elbows which are almost 0.0 mm (local perforation)—2.4 mm and the 10 years of service life until failure, the corrosion rate can then be calculated as follow^6^:

$$\text{Local Corrosion Rate}=\frac{\text{original thickness}-\text{final thickness}}{\text{service life}}$$

$$\text{Local Corrosion Rate }(\text{UB}1-\text{B}2)=\frac{6.55-0.00}{10.0}=0.66\text{ mm}/\text{year}$$

$$\text{Corrosion Rate }(\text{UN}19)=\frac{6.55-2.4}{10.0}=0.42\text{ mm}/\text{year}$$

The local corrosion rate of 0.66 and 0.42 mm/year are very high and constitutes an abnormal rate of damage of the heater piping system.

### Main findings

1. The corrosion damage occurred at the external surface of the pipes, indicating that a reaction between the heater internal atmosphere (i.e., flue gases) and the steel piping system.
2. The internal surfaces of pipes and elbows were sound and intact.
3. Corrosion occurred at the cold sides of the pipes and elbows facing the heater shell.
4. The corrosion pattern is broad and shallow pitting.
5. The damage is due to condensation of water or liquids on the piping system.
6. The pit size of UN19 and UB1–B2 average at ~ 10 mm in diameter.
7. The microstructures are typical of mild carbon steel.
8. Both of elbows UB1–B2 and the UN19 show an early stage of carbide spheroidization and pearlite decomposition. This constitutes a faulty manufacturing reason of damage.
9. The EDS microanalysis showed that the level of sulfur, chlorine and nitrogen are substantially high in the rust samples of UB1–B2 and UN19.
10. The XRD analysis of the corrosion products showed that the main oxyhydroxide which occurred is the Akaganeite.
11. The Lepidocrocite and the goethite occurred at lower levels.
12. Akaganeite occurs only chloride-laden environments. Therefore the heater atmosphere contains chlorides.
13. The elbow UN19 of the external convection section contained much lower levels of Akaganeite, and higher levels of magnetite and hematite.
14. UB10–B11 elbow does not conform to the specified composition of the SA-105 because of the low level of Mn.
15. The Si, Cu, Ni, Cr and Mo obviously exist in very low levels. These elements should be at their maximum allowable limits of the SA 105 and SA 106 to improve the corrosion resistance of the steel piping components.
16. The mechanical properties of the pipes and elbows are acceptable and do not constitute a cause for failure.
17. Minimum wall thickness reached 0.0 and 2.4 mm out of 6.55 mm in elbows.
18. The local corrosion rate of 0.66 and 0.42 mm/year are very high and constitutes an abnormal rate of damage of the heater piping system.

## Results and discussion

The corrosion damage occurred at the external surface of the investigated pipes, indicating a reaction between the heater’s internal atmosphere (i.e., flue gases) and the steel piping system. The internal surfaces of pipes and elbows were sound and intact. Corrosion occurred at the cold sides of the pipes and elbows facing the heater shell. The corrosion pattern is broad and shallow pitting. The damage is due to the condensation of water or liquids on the piping system. The pit size of UN19 and UB1-B2 average at ~ 10 mm in diameter. The microstructures are typical of mild carbon steel. Both of elbows UB1–B2 and the UN19 show an early stage of carbide spheroidization and pearlite decomposition. This constitutes a faulty manufacturing reason of damage. The EDS microanalysis showed that the level of sulfur, chlorine and nitrogen are substantially high in the rust samples of UB1–B2 and UN19. The XRD analysis of the corrosion products showed that the main oxyhydroxide which occurred is the Akaganeite. The Lepidocrocite and the goethite occurred at lower levels. Akaganeite occurs only chloride-laden environments. Therefore, the heater atmosphere contains chlorides. The elbow UN19 of the external convection section contained much lower levels of Akaganeite, and higher levels of magnetite and hematite. UB10–B11 elbow does not conform to the specified composition of the SA-105 because of the low level of Mn. The Si, Cu, Ni, Cr and Mo obviously exist in very low levels. These elements should be at their maximum allowable limits of the SA 105 and SA 106 to improve the corrosion resistance of the steel piping components. The mechanical properties of the pipes and elbows are acceptable and do not constitute a cause for failure. Minimum wall thickness reached 0.0 and 2.4 mm out of 6.55 mm in elbows. The local corrosion rate of 0.66 and 0.42 mm/year are very high and constitutes an abnormal rate of damage of the heater piping system^1^.

The corrosion damage of the piping system of a gas heater was investigated in this work. Severe corrosion occurred in about 10 years of service. Corrosion rates of 0.66 and 0.42 mm/years co-occurred in the piping system. By analysis of the circumstances of this corrosion damage, it became clear that the damage was observed in only the cold areas of the heater (areas facing the shell) and the lower parts of the piping system. The mechanism of damage is the flue gas dew-point corrosion. This damage is common in all fired process heaters operating with fossil fuels when the steel surface temperature drops to the dew point temperature. This temperature depends on the contaminants of the flue gasses. The major and substantial gases are the sulfur dioxide, sulfur trioxide and hydrogen chloride within the combustion products. The dew of these gases with water would be acids such as sulfuric acid and hydrochloric acid. These acids cause the corrosion of the steel piping^5^.

The levels of chloride and sulfide in the fuel gas are low according to the ideal data supplied by the company. However, these levels may vary with time. In addition, the high level of chloride is likely due to the saline and chloride deposits at the marine and coastal areas. The sulfur oxides are also abundant in the industrial area of the reported case study. The EDS microanalysis showed that high levels sulfur, chlorine and nitrogen do exist in the rust samples. This confirms that the flue gases contained gases of these corrosive species.

The question that arises here is that what is the dew-point temperature that must be reached in order to cause a dew condensation of liquids on the steel surfaces? The dew point of sulfuric acid depends on the concentration of sulfur trioxide in the flue gas, but is typically about 138 °C. Similarly, the dew point of hydrochloric acid depends on the concentration of hydrogen chloride. It is typically about 54 °C.

An additional question arises in this concern; were these temperatures reached during the operation of the heater? Before answering this question, it is worth noting that the inlet gas temperature is about 56 °C and the outlet gas temperature is 60 °C.

This low operation temperature constitutes favorable conditions for the condensation of liquid from the heater atmosphere. Not only this, the heater went frequently out of service because of operating conditions. During these outings heater temperature goes down and sure dew condensation of acidic liquids occurred. Therefore, the dew temperatures of sulfuric and hydrochloric acids are readily reached during the operation of the heater, especially at the cold areas^5^.

Another question arises here: how much water should be there for the corrosion damage to proceed? Is the immersion in water required? The answer as extracted from the literature is that "only very thin adsorbed film of water is all that is required for this damage to occur"^1,5^. From where does this water come? Is it from the natural humidity which is usually high in the coastal area? In addition, the water is a major product of the combustion of the natural gas fuel.

Sulfuric acid corrosion of carbon steel or low alloy steel components will have general wastage often with broad, shallow pits. At lower temperatures, hydrochloric acid may condense and promote HCl corrosion of carbon steels. The form of carbon steel damage is general uniform thinning localized corrosion or under-deposit attack. All these characteristics are observed in the current piping corrosion of the heater.

Did the material composition or metallurgical structure play any role in this failure? The material chemical composition as discussed earlier does not provide the best performance against corrosion in the service conditions of the heater. In addition, some components such as UN19 and UB1–B2 which were heavily damaged in service, exhibit a partially decomposed pearlite with an early stage of spheroidization of carbides. These elbows went through a wrong heating cycle before forming or at the heat treatment stage. These obviously went through faulty manufacturing. These metallurgical deficiencies do play a role in the early corrosion of the piping system.

Concluding, the root cause of this failure is clearly the operation of the heater at low temperatures and the frequent outings of service, combined with evident material drawbacks such as improper composition and faulty manufacturing defects.

## Conclusions and preventive actions

In the current investigation, the failure of natural gas heater pipes was investigated. The mechanism of corrosion damage was decided to be the flue gas dew point corrosion and the root cause of failure was owed to the operation of the heater at low temperatures and the frequent outings of service. This low-temperature operation takes the steel surface temperature down to the dew point temperature.

Another cause of failure is the evident material drawbacks such as improper composition and faulty manufacturing defects. The faulty manufacturing was mainly the inappropriate heat treatment of the steel by holding it at a higher temperature which produced pearlite decomposition at shorter times.

The following recommendations can be followed to minimize the flue gas dew-point corrosion of the heater:

- Use steel with the Cu, Si, Ni, Cr and Mo elements at their upper specified limits of SA 105 and SA106 Grade B, i.e., 0.4% Cu, 0.35% Si, 0.4% Ni, 0.4% Cr and 0.15% Mo. These might be specified in the material orders, beforehand.
- Make sure that the piping materials are free from faulty manufacturing defects such as carbide spheroidization and pearlite decomposition.
- Maintain the heater at some higher temperature than the current condition if the process permits.
- Minimizing the service outings.
- Remove the deposits away from the piping surfaces in a timely and periodic manner (e.g., during shutdowns).

## Data Availability

All the data generated or analyzed during this study are included in this published article.
